# Short-term effects of drought on tropical forest do not fully predict impacts of repeated or long-term drought: gas exchange versus growth

**DOI:** 10.1098/rstb.2017.0311

**Published:** 2018-10-08

**Authors:** Patrick Meir, Maurizio Mencuccini, Oliver Binks, Antonio Lola da Costa, Leandro Ferreira, Lucy Rowland

**Affiliations:** 1 Research School of Biology, Australian National University, Canberra, Australian Capital Territory 2601, Australia; 2 School of Geosciences, University of Edinburgh, Kings Buildings, Mayfield Road, Edinburgh EH9 3FF, UK; 3 CREAF, Campus UAB, Cerdanyola del Vallés 08193, Spain; 4 ICREA, Barcelona 08193, Spain; 5 Instituto de Geosciências, Universidade Federal do Pará, Belém, PA 66075-110 Brazil; 6 Museu Paraense Emílio Goeldi, Belém, PA 66040-170, Brazil; 7 Geography, College of Life and Environmental Sciences, University of Exeter, Amory Building, Exeter EX4 4RJ, UK

**Keywords:** El Niño, tropical forest ecology, forest productivity, Amazon, carbon allocation, field experiment

## Abstract

Are short-term responses by tropical rainforest to drought (e.g. during El Niño) sufficient to predict changes over the long-term, or from repeated drought? Using the world's only long-term (16-year) drought experiment in tropical forest we examine predictability from short-term measurements (1–2 years). Transpiration was maximized in droughted forest: it consumed all available throughfall throughout the 16 years of study. Leaf photosynthetic capacity 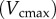 was maintained, but only when averaged across tree size groups. Annual transpiration in droughted forest was less than in control, with initial reductions (at high biomass) imposed by foliar stomatal control. Tree mortality increased after year three, leading to an overall biomass loss of 40%; over the long-term, the main constraint on transpiration was thus imposed by the associated reduction in sapwood area. Altered tree mortality risk may prove predictable from soil and plant hydraulics, but additional monitoring is needed to test whether future biomass will stabilize or collapse. Allocation of assimilate differed over time: stem growth and reproductive output declined in the short-term, but following mortality-related changes in resource availability, both showed long-term resilience, with partial or full recovery. Understanding and simulation of these phenomena and related trade-offs in allocation will advance more effectively through greater use of optimization and probabilistic modelling approaches.

This article is part of a discussion meeting issue ‘The impact of the 2015/2016 El Niño on the terrestrial tropical carbon cycle: patterns, mechanisms and implications’.

## 1. Introduction

The impacts on land of the El Niño Southern Oscillation (ENSO) include extended extremes of drying and warming, with notable climate anomalies usually seen in SE Asia, East Africa and eastern Amazonia [1]. The effects on the tropical land surface are often associated with net emissions of carbon dioxide (CO_2_) to the atmosphere, with notable peaks observed from the ENSO events of 1982/83 and 1997/98 (e.g. [2]), and the recent 2015/16 event showing strong regionally variable signals across the tropics [3]. The CO_2_ emissions anomalies associated with ENSO are frequently large, ranging up to 2.5 Pg C yr^−1^ [4]. They provide strong evidence that tropical forests exert a dominant influence on inter-annual and longer-term variations in the global flux of CO_2_ from the land to the atmosphere [5].

Measurements at smaller scales have proved consistent with these inferred atmospheric fluxes. Regional-scale airborne data have confirmed the potential for large areas of tropical forest such as those in the Amazon basin to alter the sign of their land carbon sink in response to extremes of climatic warming and drying, with at least half of the signal caused by changes in ecophysiological processes in natural ecosystems [6]. Extensive ground-based measurements of tree growth, recruitment and mortality in an Amazon-wide forest plot monitoring network have further demonstrated how extreme drought events can cause reductions in growth and increases in tree mortality, with the effects of reducing the regional net carbon sink [7], or of switching the sink to a temporary source [8].

Following early climate-driven modelling of switches in the tropical land carbon sink [9,10], studies of gas exchange at leaf and stand scales have since helped enable significant advances in understanding and simulation [11–18]. However, it remains unclear whether the ecophysiological process modelling described from short-term observations made during an El Niño or similar short-term drought equip us with enough information to understand (and then predict) the impacts of possible future repeated ENSO events [19], or of a drier, warmer climate.

The need to bridge timescales has become much stronger in view of future climate predictions of increased drought resulting from extended dry seasons and a higher frequency in extreme events, including more ENSO events, particularly in areas of Amazônia [19–23]. With a few exceptions (e.g. the responses to elevated CO_2_), simulations of future land–atmosphere interactions necessarily tend to make the assumption that process representation derived from short-term observations can be extrapolated to simulate responses at multi-annual and multi-decadal timescales. However, fundamental limitations in ecological understanding exist with respect to connecting our understanding of processes across timescales, particularly from seasonal and annual scales (which tend to be fairly well studied) to decadal scales and beyond [24]. Beyond short-term (less than 1 year) responses in, for example, gas exchange by leaves and soil, additional ecological responses to drought may need to be accounted for, such as changes in phenology, reproductive output, carbon allocation to growth above- and below-ground, differential tree mortality and ultimately changes in taxonomic composition and their impacts on community-level functional trait distributions and overall functioning (e.g. [25,26]). Understanding these changes and then accounting for them in a model require an adequate (and parsimonious) series of connections to be made between the acquisition of carbon by trees and its diverse and changing metabolic destinations. Chronosequence studies can only partially inform this question; the only way to test the responses to a direct climate perturbation such as drought is to use experimental manipulation [27].

Only three such multi-year ecosystem-scale ‘drought’ experiments in tropical rainforest have been published to date, two in Amazonia and one in Sulawesi [28]; a fourth is in progress, in NE Australia [29]. The Amazonian experiments have generated multi-annual (greater than 5 years) datasets [30,31], and one of these, examined here, has reported decadal-scale data during which longer-term ecological processes have become quantifiable [28,32]. Both the field data from the Amazon and Sulawesi experiments, and initial testing of dynamic vegetation model performance against growth and mortality data, have identified the need for better connections between soil and plant hydraulics, and tissue structure [14,17,18,32–34]. Most notably, the inclusion of plant water potential and plant trait-based constraints determining the limits to water transport in woody tissue have begun to be used to link drought stress with tree mortality; Eller *et al*. [35] report on one such model development.

The mismatches between model performance and observed long-term responses to drought underline fundamental uncertainties over whether a simple accumulation of short-term responses or qualitatively distinct response modes determine ecological change over time. For example, can observations of declines in photosynthesis or growth made during short-term drought (e.g. [30,36–38]) predict the response to repeated or longer-term climatic drought? The answer is key to assessing how reliable multi-decadal model predictions might be (and their importance for environmental policy), but few data are available to constrain the large inherent uncertainty over whether long-term stabilization or continued rapid biomass decline can be expected [24,28,39–41].

Vegetation models generally describe a principal response to climate, consistent with the idea of resistance [42]: climate alters photosynthetic supply to growth leading to declines or increases in performance. In reality however, an initial reduction may be followed by recovery in one or more component processes; or stem density and species composition may change, with functional effects at individual and community levels. Recovery in a process may denote resilience to the climate stress, perhaps also including wider coordinated changes in growth, reproduction and mortality. Short-term examples of such switches in response to drought that could not be predicted purely from a change in gross productivity include mast flowering responses in SE Asian Dipterocarps (e.g. [43]) and the prioritization of stem growth in SW Amazonia [44,45]. The lack of consistent mortality and growth responses in Amazonian trees following two near-consecutive severe regional drought events in 2005 and 2010 [7,8] illustrates the need for new insight. Here, high mortality but a smaller overall reduction in growth in the 2005 drought was followed by a smaller mortality signal but a larger reduction in growth during the 2010 drought [7]. These differences in growth and mortality might merely have resulted from spatial and temporal variation in climate, plant hydraulic vulnerability and gross photosynthesis, or alternatively, additional growth responses may have played important roles.

Here we use the leverage of the only decadal-scale rainfall exclusion experiment in tropical forest to test whether observations made over minutes to months can be used to predict changes in ecological function at longer timescales. Field-based drought experiments are implemented by deflecting away from the soil a fraction of the rainfall that penetrates the canopy (‘throughfall exclusion’, TFE), thus increasing soil moisture deficit [28,46]. This manipulation separates the influences of soil and atmospheric drought on vegetation processes, but it also results in less extreme stress than a natural drought would exert with similarly low rainfall, as the maxima of air temperature and vapour pressure deficit are smaller. Thus, while the long experimental time-series in this study provides particular insight, we acknowledge that more extreme or more rapid responses to ENSO-related or other drought events could occur. We focus our analysis on processes at different ecological scales, but all cover more than a decade of experimental soil moisture deficit: leaf photosynthetic capacity, sap flux, litterfall, tree growth and mortality, and soil respiration. Our goal is to test whether long- and short-term (e.g. ENSO-related) responses to drought can be treated as similar or whether they differ qualitatively, with consequences for model representation. We pose a single hypothesis and examine it with respect to each metric in turn: ‘the response to soil moisture deficit is similar at short (1–2 year) and decadal timescales’.

## 2. Methods

The site of the long-term through-fall exclusion (TFE) experiment is the Caxiuanã National Forest Reserve in the eastern Amazon, Pará State, Brazil (1°43′ S, 51°27′ W). Rainfall is 2000–2500 mm yr^−1^, with a pronounced dry season (June–November, rainfall less than 100 mm month^−1^). It is situated on *terra firme* forest, with yellow oxisol soils [47].

The TFE experiment consists of two 1 ha plots located in old-growth forest. The treatment (TFE) plot has been covered with plastic panels and guttering placed at 1–2 m above the soil since January 2002. This structure excludes approximately 50% of the incoming canopy through-fall from the soil. A ‘Control’ plot on which no rainfall exclusion has taken place is located less than 50 m away. For details on the experimental design, see [28,31].

Multiple measures of growth and physiology have been made on the trees and soil during the experiment [28]. The metrics and source studies used here are summarized below. Continuous or semi-continuous datasets have been recorded for on-site meteorology and soil moisture content, growth, mortality and recruitment of all trees above 10 cm stem diameter [31,32], and for litterfall [48]. Most other metrics have been obtained in campaign mode, with intensive periods of measurement at the outset of the experiment and more recently, from 2012. Soil properties and CO_2_ effluxes are reported in [47,49–51]. Soil CO_2_ efflux (soil respiration, *R*_soil_) sampling was made using the closed-circuit infrared gas analyser method [49]. Temperature-corrected leaf photosynthetic capacity 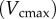 measurements followed standard procedures [13], with 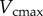 derived from full *A/C*_i_ response curves. Measurements of 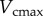 were made seasonally during 2001/2003, and then during 2013/2014 [32], with recent intensive data from 2016 (L Rowland *et al*., unpublished data). Sap flux measurements were made using the same heat-balance method in both periods [14,52,53]. The sampling of trees and species for physiology and sap flux measurements was guided by canopy access at the start of the experiment, and subsequently by mortality data [31], which enabled the identification of species with high and low mortality responses to the TFE treatment that could be then be sampled with replication in focused ecophysiological studies. Additional measurements that have been made but are not considered in detail here include: hydraulic vulnerability of xylem tissue and the concentration of non-structural carbohydrate in leaves, stems and roots [32]; leaf water relations and cellular structure [18,34,54]; and the flux of CO_2_ from leaves and woody tissue [55–57].

Reported values and error terms use those presented in the original publications. Our comparative framework tests whether drought responses observed over the early phase of the TFE can be used to reliably predict those same responses over the long term and therefore suggesting simple and reversible resistance to moisture stress, or whether we observe more complex behaviour including resilience in some ecological metrics over the long-term.

## 3. Results

### (a) Ecophysiology of leaves, soil and canopy

**Figure 1.**
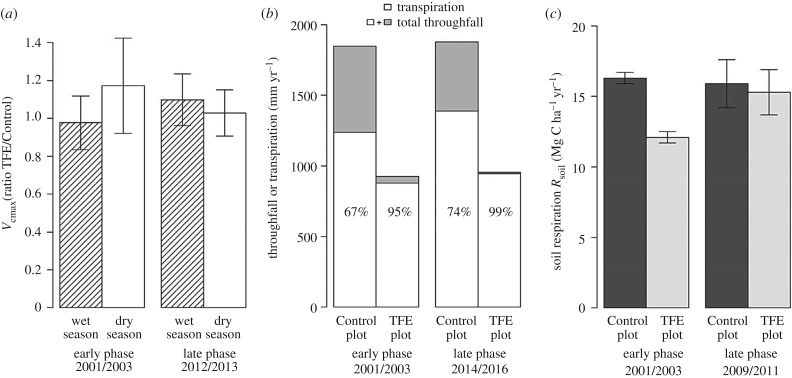
The changes over short (1–2 year) and long (decadal) timescales in: (*a*) ratio of foliar photosynthetic capacity (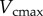 at 25°C) in TFE and Control plots, in wet and dry seasons; (*b*) annual throughfall and sap flux totals in TFE and Control plots; and (*c*) annual soil respiration totals (*R*_soil_) in TFE and Control plots. The data compare the effects on each variable of experimental soil moisture reduction, imposed by throughfall exclusion (TFE) compared with non-droughted, Control forest. The time periods compared are from 2001/2003 to 2012/2013 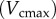, 2014/2016 (sap flux) and 2009/2010 (*R*_soil_). (*a*) 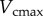 is plotted as the ratio of TFE/Control in the wet and dry seasons (actual 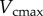 values, e.g., from 2012/2013 are 29.1 ± 1.8 s.e. µmol m^−2^ s^−1^, Control and 26.8 ± 1.6 s.e. µmol m^−2^ s^−1^, TFE). (*b*) Total throughfall (shaded) and sap flux (open) values are annual fluxes (mm yr^−1^; see §2); the % values quantify the % of total throughfall that is recycled by transpiration in each plot and time period. (*c*) *R*_soil_ (t C m^−2^ yr^−1^ ± s.e.) are annual totals based on regular chamber-based flux measurements in each period, compared across TFE and Control plots. All data are re-expressed from [14,32,49,51,53,57].

Our photosynthetic capacity data have been limited by the total number of species measured and the goal of replication of species showing high and low mortality rates in response to drought (15 species in 2001/2003 and 10 species in 2013/2014) [55]. In this work, 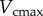 showed no significant change under drought (TFE treatment) in comparison to the un-droughted (Control) forest (figure 1*a*). However, over time, mortality opened up the canopy, changing competition for resources and significantly increasing light availability to some trees. Recent intensive sampling of 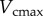 shows the mean 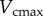 of sunlit canopy leaves across all tree sizes to remain unchanged, consistent with previous findings, but where tree crowns have become fully exposed to light, some downward acclimation of 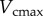 is observed. By contrast, the soil respiration flux (*R*_soil_) declined immediately in response to the experimental soil moisture deficit during 2001/2003, reaching a reduction of more than 20% [49], but later showed evidence of long-term recovery, with *R*_soil_ in the TFE returning to near that of the Control forest after four years (figure 1*c*) [50,51,57].

A short- and long-term comparison of leaf stomatal conductance is not yet available, but tree-level sap flux data were obtained during both 2001/2003 and 2014/2016. A significant relationship (*p* < 0.01) was observed between stem diameter and maximum sap flux (the average daytime wet-season flux, kg cm^−1^ h^−1^) over a large range in stem sizes, 5–70 cm diameter [14,53]. This relationship was used to model annual water use together with: inventory data for each plot; sap flux measurements in both dry and wet seasons from 2014 to 2016; and accompanying meteorological and soil moisture drivers. Transpiration was 68–71% lower in the TFE than the Control (879 versus 1238 mm yr^−1^ in 2002/2003 and 945 versus 1389 mm yr^−1^ in 2014/2016 (figure 1*b*)). In each period, very close to 100% of the rain-fed water available after TFE was recycled as transpiration, significantly more than the 67–74% recycling of normal rainfall performed by non-droughted Control forest (figure 1*b* and [53]). While light and vapour pressure deficit drove daily sap flux in both forest plots, soil moisture availability mainly determined transpiration in the TFE in the dry season, but was not a constraint in the wet season [14,53,58]. Leaf area index in the TFE was reduced by 12–20% during 2001 to 2015 [14,32], although estimates of leaf area index contain measurement uncertainty [59]; this implies slightly higher leaf-level stomatal conductance during wet season release from drought. However, the principal cause of the reductions in stand-scale transpiration in the TFE differed over time. In 2001/2003, at high biomass and presumably more intense competition for water, stomatal control at the leaf level strongly reduced canopy water use [14,60]. During 2014/2016, however, stand-scale water use was mainly controlled by the reductions in sapwood area that had resulted from high preceding mortality during the experiment [53]. The high recycling rate (approx. 100%; figure 1*b*) observed during 2014/2016 was facilitated by markedly increased wet-season rates of transpiration in the surviving trees, consistent with higher stomatal conductances; conversely, dry season transpiration rates were very low, constrained by low soil moisture availability.

### (b) Biomass change, tree mortality and litter production

**Figure 2.**
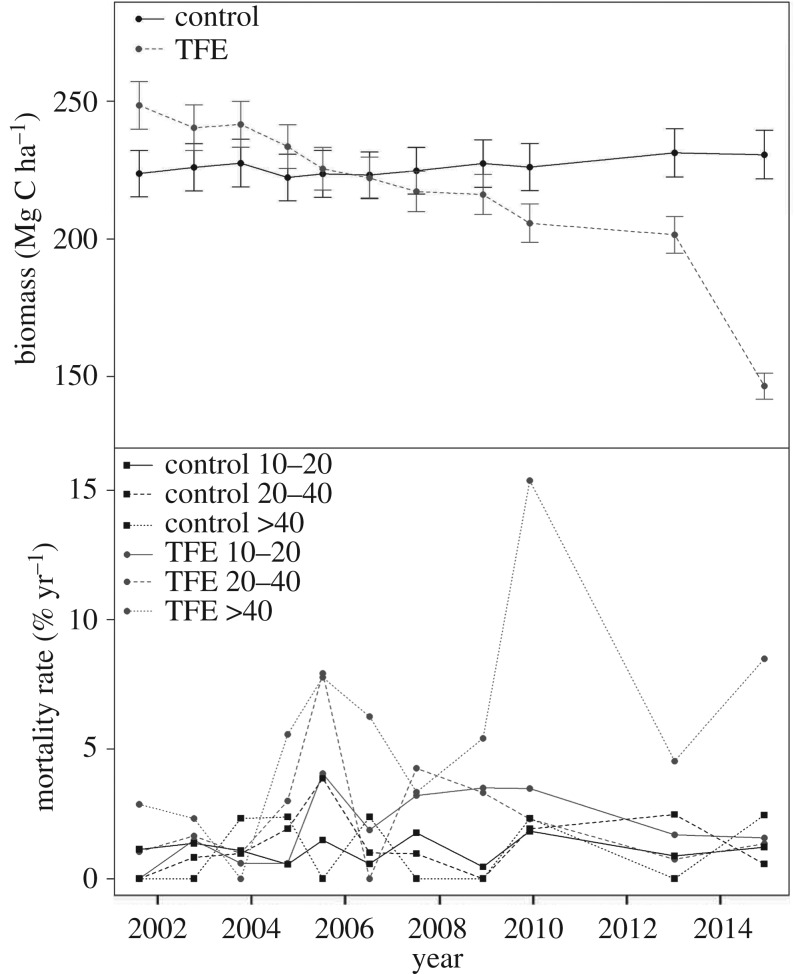
Changes in biomass and mortality rates in response to 14 years of experimental soil moisture reduction imposed by throughfall exclusion (TFE, grey), compared with non-droughted forest, ‘control’ (black). (*a*) Biomass change from 2001 to 2014 (Mg C ha^−1^ yr^−1^). Error bars are s.e. over 12 measurements, accounting for local woody tissue density and allometry. (*b*) Mortality rate (% stems per year) of trees of 10–20 cm stem diameter (control *n* = 164–193, TFE *n* = 132–174, with range showing 2001/2014 maximum and minimum *n*), 20–40 cm stem diameter (control *n* = 97–105, TFE *n* = 81–104) and greater than 40 cm stem diameter (control *n* = 41–45, TFE *n* = 17–37). From Rowland *et al*. [32].

Increased tree mortality rates in response to the experimental drought were not observed for the first three years after the TFE began in 2002, but after this point mortality rates increased, with large peaks in 2005 and 2010. Increased mortality was strongly associated with the largest tree size group (stem diameter greater than 40 cm; figure 2). Overall, mortality and related biomass loss were substantially higher in the droughted TFE forest, with a loss of nearly 20% biomass by 2008, accelerating to an overall loss of 40% by 2015 (figure 2) [31,32]. Woody tissue production declined during the first seven years of the TFE by 30% relative to the Control forest, but only in the medium and large stems (diameters 20–40 cm and greater than 40 cm), with little growth reduction in the smaller stems (diameter 10–20 cm) [31]. From year eight, after initial mortality and associated changes in resource availability, especially light and moisture, TFE tree growth rates recovered relative to the Control [32], with higher rates than in Control for small and medium trees (10–20 cm and 20–40 cm diameter), and similar rates in the largest trees (greater than 40 cm).

**Figure 3.**
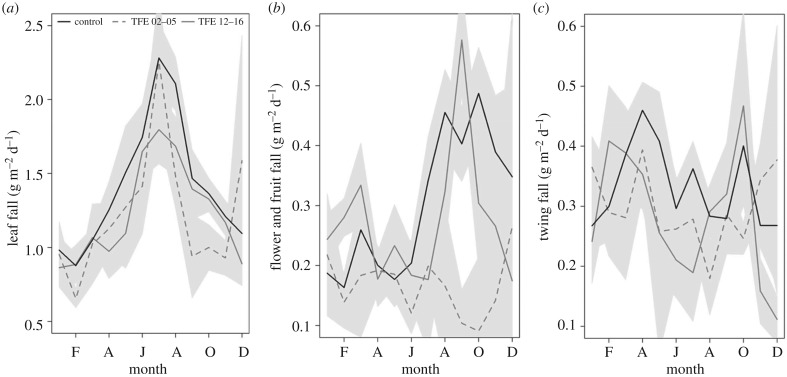
Changes in litterfall in response to 15 years of experimental soil moisture reduction imposed by throughfall exclusion (TFE) in comparison with non-droughted Control forest. (*a*) Mean daily leaf fall (g m^−2^ d^−1^); (*b*) flower and fruit fall (g m^−2^ d^−1^); and (*c*) twig fall (g m^−2^ d^−1^) for the Control plot (2001/2016, black lines), the first 4 years of the TFE (2002/2005, grey dashed lines) and the final 5½ years of the TFE (2012/2016, grey solid lines). Grey-shaded areas indicate the standard error around the lines. From Rowland *et al*. [48].

**Figure 4.**
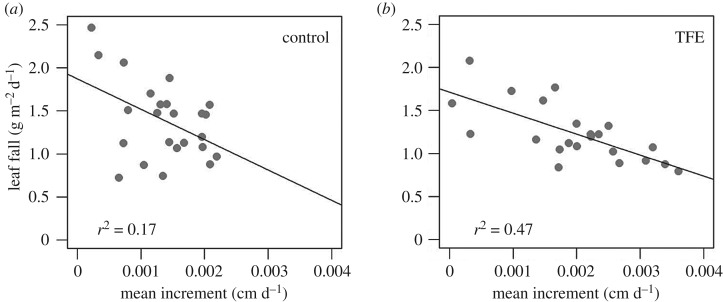
Linear correlations of mean woody growth increment with leaf fall after long-term throughfall exclusion (TFE) in comparison with non-droughted Control forest; the data are from 2010 to 2016. Increment measurements were made every three months (cm d^−1^). Leaf fall data were taken fortnightly; log leaf fall data are shown for (*a*) Control (g m^−2^ d^−1^) and (*b*) TFE (g m^−2^ d^−1^). A linear fit is shown if a correlation is significant at the *p* < 0.05 level, and the correlation coefficients for these linear lines are printed in bottom left-hand side of the panels. From Rowland *et al*. [48].

The production of leaves, flowers and fruits, and fine wood (twigs) all declined in response to the experimental drought in the first 3 years of TFE, although fine wood litterfall showed high variance and little pattern (figure 3*a*–*c* and [48]). A sharp decline (greater than 50%) in fruit and flower production occurred in the first year of TFE, compared to a smaller decline in leaf litter of 10–20%. The normal pattern of an early dry-season peak in flower- and fruit-fall was entirely lost during the early phase of the experiment (figure 3*b*). However, after 10 years of experimental drought, reproductive output recovered, and the original seasonal pattern in fruit- and flower-fall also re-established itself (figure 3*b*). The declines in leaf litterfall during the experiment were less marked, ranging 10–20%, with little disruption of the seasonal pattern of litterfall (figure 3*a*). However, after the long-term drought treatment, a strong negative correlation between leaf litterfall and mean tree growth increment was observed, showing a tighter trade-off in the TFE between these two principal above-ground production terms (figure 4) than found in non-droughted Control forest.

## 4. Discussion

The results presented here from the only long-term drought experiment in tropical forest suggest that forest function during long-term drought (greater than 10 years) is largely not predictable simply from the effects observed over the short-term (1–2 years). Stand-scale transpiration was the main exception and proved to be the most predictable flux. It was maximized throughout the experiment, although the main constraining drivers changed from physiological at the leaf and tree scale (stomata) to structural at the stand-scale (reduced sapwood area). Photosynthetic capacity of sunlit canopy leaves appeared relatively constant over time when averaged across all tree sizes, with later indications of downward acclimation to combined high light exposure and drought, following mortality-related impacts to the overall TFE canopy light environment. However, the remaining responses, in mortality, growth and reproduction, showed more complex behaviour, altering over time to reflect both resistance and resilience; they could not be predicted either from their own early-phase behaviour, or from simple changes in gas exchange activity.

Increased complexity in models and observations could account for these phenomena, but the introduction of simplifying analytical approaches that assume specific trade-offs in the components of plant production in order to maximize plant fitness may prove more effective [24,61,62]. At both experimental and regional scales there is evidence that specific wet-affiliated genera bear a higher mortality risk than others during drought [26,28,31], meaning that the process-based responses described here will be both accompanied by future changes in species abundances, and affected by any consequent changes in stand-scale functioning.

### (a) El Niño Southern Oscillation climate and the effects of experimental drought

The El Niño-driven climate anomalies that are important here comprise extended periods of reduced rainfall and soil moisture availability, and increases in solar radiation, air temperature and vapour pressure deficit [1]. By contrast, the experimental (TFE) drought manipulation has a single main initial effect: a large reduction in soil moisture availability [28]. Over the longer term this is coupled with the effects of high mortality [31,32] causing alterations to canopy structure and related availability of light, water and nutrients, which influence functioning at tree and/or stand scales. The TFE treatment thus enhances natural climatic extremes in low rainfall and high vapour pressure deficit, first imposing significant hydraulic limitations on plant function and then additional longer-term changes in resource availability and competition caused by alterations in overall forest structure. However, because it does not impose a continuous atmospheric drought in addition to that of the soil, the TFE treatment enables trees to recover partially from naturally co-occurring drought cycles (e.g. ENSO).

### (b) Ecophysiological responses

Overall, the observed biophysical controls over transpiration are consistent with water use always proceeding to a maximum however this is achieved, as long as the gains from assimilation outweigh losses from hydraulic constraints [63,64]. The TFE plot consistently used close to 100% of the available throughfall reaching the soil (figure 1*b*), a substantially higher rate than observed for Control forest (70%). Despite this constancy in maximum water use, the dominant controls on transpiration changed from individual-level stomatal control during the early, high-biomass phase of the experiment, to the reduction in sapwood area resulting from preceding mortality imposing stand-scale structural constraints on transpiration [53]. Thus, long-term transpiration is predictable from short-term measurements, at least to the extent of the current levels of structural change (i.e. 40% biomass loss). However, further substantial loss of sapwood area through mortality might reduce the capacity of the ecosystem to recycle the 900–1000 mm (approx. 50% rainfall) it currently receives. Uncertainty over whether continued TFE treatment will cause further biomass collapse or lead to a new equilibrium state therefore challenges our understanding of the response to drought at multi-decadal scales.

The *average* maximum photosynthetic capacity 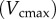 of sunlit canopy leaves across all trees sizes was relatively predictable from short to longer timescales, with no significant decline throughout the experiment [32], even after more than 10 years of reduced soil moisture availability (figure 1*a*). The metabolic cost of maintaining high 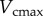 during drought stress thus appears—on average—to have been compensated for (*sensu* [65]) by enabling high net assimilation rates when water becomes available, for example early in the morning or following episodic rain. However, *in extremis*, drought-acclimation of 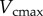 does ultimately occur in full light-exposed and long-term droughted tree canopies, following high mortality and related structural change, with potential effects on gross productivity via tree-scale differences in canopy illumination and performance.

The sharp initial decline in soil respiration, *R*_soil_ (figure 1*c*) [49], was consistent with wet-to-dry season moisture-response functions for *R*_soil_ in other tropical forests (e.g. [66]). However, after four years of TFE this response pattern became disrupted, with *R*_soil_ fluxes in the droughted TFE forest moderated back upwards to 95% of that in non-droughted Control forest [50,51]. The long-term signal here may have reflected higher live root respiration rates associated with co-incident increases in specific root length and fine root production, despite likely lower root density [57,67] following mortality and root decomposition [30]. Overall, figure 1*c* suggests that *R*_soil_ is not predictable during long-term drought solely from short-term responses because of switches in metabolism and growth patterns.

### (c) Tree mortality and net primary production

The initial 3-year resistance to mortality in this experiment [28,31] contrasts with observations of more rapid increases in mortality during single-year natural drought events (e.g. [8,68,69]). This difference likely reflects the lack of high vapour pressure deficit and air temperatures in the experiment that otherwise accompany a natural severe drought. However, at least for the Amazon, forest plot monitoring data for the repeat natural droughts of 2005 and 2010 have shown that differences in mortality and reductions in growth do not simply reflect immediate drought severity or prior mortality incidence: they also vary with preceding climate, particularly soil moisture availability [7]. Consistent with this, significant increases in mortality in this experiment only occurred below a threshold of a relative-available-soil-moisture of 50%, irrespective of the time since the TFE drought treatment began [28].

The plant physiological determinants of drought-related tree mortality have been widely discussed and reviewed elsewhere [17,24,28,32,70,71]. Acknowledging the difficulty of complete understanding of the mortality process [63], an increasing consensus has built towards advancing dynamic vegetation models by connecting plant water potential with the hydraulic vulnerability of xylem tissue [32,71]. This approach makes assumptions of optimal behaviour (e.g. [35]) and has the advantage of simplifying model structure with respect to soil moisture supply and stomatal conductance by using the same physical processes to determine changes in water loss, assimilate supply and overall growth and mortality, over both short and long time scales.

The mortality patterns in the experiment changed between short and longer timescales, but with further testing both may prove to be explicable using these modelling approaches, by combining new representation of changes in plant water potential during drought with better understanding of associated mortality thresholds. The substantially different stand dynamics observed in both experimental contexts [24,28] and during different natural droughts, such as the 2005 and 2010 Amazonian events [7], may thus ultimately be accounted for without recourse to more complex changes in allocation and growth.

As reported for forests globally [72], the size class most sensitive to drought was those trees greater than 40 cm in stem diameter (figure 2*b*). Consistent with this, tree growth rates declined at the start of the experiment in the largest but not the smallest trees [31], and following increased mortality during 2005/2012, competitive release and altered resource availability led to the recovery of growth rates in the remaining larger trees, and to increased growth in the smaller trees relative to that in non-droughted forest [32]. This plasticity in growth over time suggests that long-term growth responses cannot be predicted simply from short-term data, though we note that models accounting for changes in resource availability (light, soil moisture) following mortality may ultimately be capable of simulating the observed resilience in growth.

As observed elsewhere in long-term tropical forest monitoring studies [73], differences in allocation to reproduction, foliage and fine woody litterfall varied over time [48]. The sharp early decline in fruit- and flower-fall followed by a recovery described clear resilience in reproductive output (figure 3). The stronger correlation between leaf litterfall and stem growth following long-term drought than in Control forest (figure 4) also pointed to a key trade-off between allocation to growth sinks in the canopy and stem. Here, the tighter relationship between them under drought (figure 4*b*) likely emerged because of constrained overall availability of non-structural carbohydrate. The responses observed in reproductive or leaf litterfall (figures 3 and 4) could not have been predicted from their short-term responses (or simple declines in gross assimilation) and, similar to the sap flux and growth data, they likely resulted from long-term changes in resource availability following mortality.

## 5. Conclusion

We summarize our findings by mapping back to the framework of examining how predictable long-term responses are from short-term behaviour, and by considering how this can inform future modelling of the responses by tropical forests to individual (e.g. El Niño), repeated or long-term droughts.

### (a) Can short-term responses to drought be used to predict future impacts?

**Figure 5.**
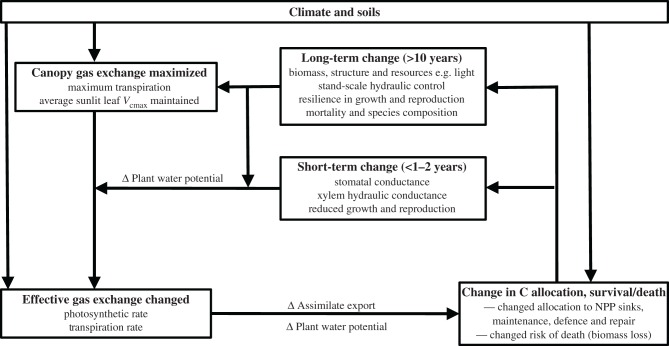
A schematic of the responses to drought over 1–15 years, based on a throughfall exclusion (TFE) drought experiment in tropical rainforest. Changes over short- (less than 1–2 years TFE) and long-term (10–15 years TFE) timeslices emphasize the long-term effects of mortality on changed resource availability and the response by vegetation in different metrics. Climate and soils provide both background and immediate constraints on photosynthetic capacity, potential transpiration, actual photosynthesis and transpiration, and on the allocation of carbon to metabolism for net primary production (NPP, i.e. net growth), reproduction, maintenance, defence. Only transpiration is maximized across the short- and long-term, using all the water available (100% recycling of throughfall). Photosynthetic capacity 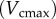 is maintained when averaged across tree size groups (though downward acclimation occurs in very light-exposed canopies; see §4b). Actual rates of transpiration and photosynthesis are modified rapidly according to immediate plant water potential and microclimate drivers, with total transpiration constrained at high biomass (short-term) by stomatal control and imposed by low sapwood area at the stand scale, following long-term structural changes from tree mortality. Actual gas exchange rates are optimized by altering, and being affected by, plant water potential and assimilate export. Differential allocation of assimilates to metabolic outcomes is effected through multiple trade-offs (presumed) to maximize individual fitness, leading to resilience in some metrics (e.g. growth, reproduction). Changes in reproduction and mortality affect species composition and structure, influencing resource availability to individual trees, altering local light and soil moisture availability and average plant tissue hydraulic properties, further influencing plant water potential and resultant assimilation and its allocation.

The predictability of long-term response modes from short-term behaviour fell into categories of increasing complexity, combined in figure 5. (i) Transpiration was maximized at all timescales: in droughted forest it was approximately 70% of that in non-droughted forest, but 100% of the rain-fed water available to the droughted forest was always used by the forest in transpiration. (ii) The 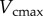 of sunlit leaves, which directly influences gross photosynthesis, remained constant from the short- to the long-term when *averaged* across all trees, although recent intensive sampling suggests limited downward acclimation of 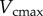 in fully light-exposed and droughted tree crowns, following preceding mortality. (iii) Increased mortality incidence was not predictable from short-term responses alone, but was strongly associated with tree size, and may be predictable over multiple timescales with emerging new model representations of plant and soil hydraulics. (iv) The long-term patterns in net primary production and respiration were also not predictable from short-term responses to drought. Instead they appeared dependent on how the dynamics of resource availability (light, moisture) following mortality affected trade-offs among allocation to stems, reproduction, leaves and roots, or to metabolic demands associated with stress and tissue repair (figure 5). Observations of changes in allocation favouring stem growth have been observed elsewhere in tropical forest during short-term natural drought [44,45], so improved understanding of allocation rules in response to climate stress may be needed over short timescales as well as long.

### (b) Consequences for modelling the response to drought by tropical forests

There is current intensive focus on the incorporation of the physical principles of soil and plant hydraulics, and plant water potential, into new model frameworks (e.g. [24,35,74]), and on the measurement of related plant traits, particularly plant hydraulic vulnerability [32,75]. This will advance our ability to account for drought-related patterns in tree function and mortality over multiple timescales, but we also suggest that improved modelling of soil water availability will prove to be a critical addition to this effort. Little information is available on this: much more extensive monitoring, measurement and modelling are needed [76–79].

We also highlight the importance of dynamic long-term drought impacts on forest structure and its consequences for resource availability to surviving trees following mortality. Simulation of the effects of repeated ENSO-like climate anomalies or long-term drying will need to account for potentially complex, perhaps cumbersome, changes in computed competition for light, water and nutrients. However, more efficient ways forward are emerging. For example, the differences in growth and mortality observed in the 2005 and 2010 Amazon droughts [7] may be predictable through new optimality-based simulations of soil and plant hydraulics [74]. Similarly, alterations to the allocation of assimilate among growth or other metabolic sinks, and their impacts on carbon residence times and net carbon balance (cf. [41]) may also be addressed more effectively by using goal-seeking formulations in models, such as optimization or probabilistic approaches, rather than only by adding multiple new empirically-derived process representations [28,62,80,81]. Introducing such newer modelling approaches [24,61,82] into existing mechanistic model structures should accelerate improvements in our understanding and prediction of the far-reaching effects of drought on tropical forests.

## Data accessibility

This article has no additional data.
